# Chemically Engineered Titanium Oxide Interconnecting Layer for Multijunction Polymer Solar Cells

**DOI:** 10.3390/polym16050595

**Published:** 2024-02-21

**Authors:** Geunjin Kim, Hyungcheol Back, Jaemin Kong, Laiba Naseer, Jiwon Jeong, Jaehyoung Son, Jongjin Lee, Sung-Oong Kang, Kwanghee Lee

**Affiliations:** 1 Hanwha Solutions, Seoul 04541, Republic of Korea; geunjin.kim@qcells.com (G.K.); hyungcheol.back@qcells.com (H.B.); 2 Department of Physics, Research Institute of Natural Sciences, Gyeongsang National University, Jinju 52828, Republic of Korea; naseer.laiba@gnu.ac.kr (L.N.); zone@gnu.ac.kr (J.J.); sonfix@gnu.ac.kr (J.S.); bandy1@gnu.ac.kr (J.L.); 3 Department of Chemical Engineering, Hanyang University, Ansan 15588, Republic of Korea; 4 MExplorer Co., Ltd., Ansan 15588, Republic of Korea; 5 Department of Materials Science & Engineering, Gwangju Institute of Science and Technology, Gwangju 61005, Republic of Korea; 6 Heeger Center for Advanced Materials, Gwangju Institute of Science and Technology, Gwangju 61005, Republic of Korea

**Keywords:** multijunction solar cells, interconnecting layers, chemically engineered titanium oxide

## Abstract

We report chemically tunable n-type titanium oxides using ethanolamine as a nitrogen dopant source. As the amount of ethanolamine added to the titanium oxide precursor during synthesis increases, the Fermi level of the resulting titanium oxides (ethanolamine-incorporated titanium oxides) significantly changes from −4.9 eV to −4.3 eV, and their free charge carrier densities are enhanced by two orders of magnitudes, reaching up to 5 × 10^18^ cm^−3^. Unexpectedly, a basic ethanolamine reinforces not only the n-type properties of titanium oxides, but also their basicity, which facilitates acid–base ionic junctions in contact with acidic materials. The enhanced charge carrier density and basicity of the chemically tuned titanium oxides enable multi-junction solar cells to have interconnecting junctions consisting of basic n-type titanium oxides and acidic p-type PEDOT:PSS to gain high open-circuit voltages of 1.44 V and 2.25 V from tandem and triple architectures, respectively.

## 1. Introduction

As the power conversion efficiency (PCE) of organic solar cells (OSCs) approaches and surpasses ~18% in a single junction architecture [1,2,3], great attention has been focused on multi-junction connections that can further enhance either the voltage or current of solar cells in series or parallel architecture [4,5]. Most recent multi-junction device studies are based on series-connected cells, and major investigations of connected cells have been performed on the interconnecting junction (ICJ) that physically and electrically connects component subcells [6,7]. In serially connected cells, the ICJ normally consists of two charge selective layers, and the contact of the two interconnecting layers (ICLs) forms a junction that allows for the recombination of electrons and holes collected from subcells. In ideal series-connected cells, high open-circuit voltage (*V*_OC_) corresponding to the sum of *V*_OC_s of subcells can be achieved. In reality, however, a voltage loss often occurs if a depletion layer is too thick for electrons and holes to recombine with no energy loss or an ICL depletes the counterpart ICL of its free charge carriers, where the depleted ICL loses its electrical properties. Thus, both ICLs should possess high free charge carrier densities for securing a narrow barrier or an ohmic contact at the ICJ so as to avoid a voltage loss and/or an S-shaped kink found at around *V*_OC_, which represents the high series resistance of multi-junction solar cells.

In conventional multi-junction architectures, the ICJ is conventionally composed of a sequential stack of n-type and p-type materials [7,8]. To achieve low resistance ohmic contact in the ICJ, both n- and p-type materials should be highly doped or semi-metallic. In solution-processed multi-junction cells, one more prerequisite must be satisfied, which is a solvent orthogonality to organic solvents, e.g., toluene, chlorobenzene, and chloroform, which are usually used to dissolve photoactive materials [8]. Since the second photoactive layer for a rear cell is cast on ICLs followed by the first photoactive layer for a front cell, ICLs must resist the organic solvents used to prepare photoactive solutions. For this purpose, poly(3,4-ethylenedioxythiophene):polystyrene sulfonate (PEDOT:PSS), which contains aqueous polyelectrolyte PSS, is generally employed as a p-type hole-transporting ICL. PEDOT:PSS serves as an organic-solvent-resisting layer, as well as a great p-type ICL, since PEDOT:PSS consists of oxidized PEDOT polymer chains that can carry a great number of positive charge carriers and aqueous polystyrene-based PSS polymer chains functionalized with sulfonyl groups that can resist and block the organic solvents. Since the PEDOT is synthesized via oxidative polymerization and stabilized with acidic polyelectrolytes (PSS) [9], PEDOT can be considered a highly doped p-type or even semi-metallic polymer depending on its oxidation level, but, at the same time, the aqueous PEDOT:PSS should be acidic in order to retain its oxidation state. Thus, n-type ICLs undergo proton attacks or oxidations during the solution processing of acidic PEDOT:PSS on n-type ICLs.

There are many n-type metal oxides, but only a few metal oxides can endure acids. Titanium oxide is an acid-resisting metal oxide, and its native oxygen vacancies play a role in electron donors, making the metal oxide a natural n-type semiconductor [10,11,12]. Despite the aforementioned merits, using titanium oxide as the n-type ICL in multijunction solar cells has been a challenge. There are a few successful reports where titanium oxide is used as an n-type ICL in tandem solar cells [11,13], but a successful demonstration probably requires a very narrow processing window [14], leading to a poor reproducibility that is mostly attributed to the difficulty in the free charge carrier density control of n-type titanium oxide. Thus, some groups circumvented the issue by employing another metallic layer such as ultrathin aluminum or a silver layer either before an n-type ICL or in between n- and p-type ICLs to promote an ohmic-like (or tunneling) junction [15,16,17], but these approaches also require the precise control of the ultrathin metal layers and additional vacuum processing steps for the layers.

Recently, quite successful results came out from vacancy-controlled ${\mathrm{TiO}}_{2-x}$ (where the subscript *x* represents the oxygen vacancy) with no metal layer between ICLs, achieving a high power conversion efficiency (PCE) of 20.27% in a tandem architecture [5]. This work is full of suggestions, where, in particular, the vacancy control is a crucial factor for titanium oxide to perform a role in a perfect n-type ICL, but, at the same time, it implies that the vacancy level control should be still conducted in a very sophisticated manner using electron beam evaporation; for instance, the best efficiency is attained only at ${\mathrm{TiO}}_{2-x}$ where *x* = 0.24, and a slight change in stoichiometry results in a significant drop in PCE by almost one-third. Despite their great implications, controlling the n-type properties of the metal oxides only with oxygen vacancies is still challenging, requiring further practical investigations to find a reliable working window of vacancy contents in the metal oxides, which might be quite pricey and time-consuming.

Here, we report chemically tunable n-type titanium oxide where ethanolamine (EA) is employed as a nitrogen source that might act as the electron donor in the metal oxide medium. As the amount of EA increases during synthesis, the Fermi level (E_F_) of the resulting ${\mathrm{TiO}}_{2}$ (EA-incorporated ${\mathrm{TiO}}_{2-x}$; in short, EA-${\mathrm{TiO}}_{2-x}$) decreases from −4.9 eV to −4.3 eV. The high lying E_F_ of EA-${\mathrm{TiO}}_{2-x}$ can be a great match to the lowest unoccupied molecular orbital (LUMO) levels of electron acceptors in front cells, and also gives a great difference to the E_F_ of p-type ICL, PEDOT:PSS (~5.0 eV). Moreover, the basicity of EA (p*K*a ≈ 9.50) compensates for the high acidity of PEDOT:PSS (pH ≈ 2), facilitating acid–base ionic junctions at the interface of ICLs, which not only prevents the further intrusion of protons sourcing from acid PEDOT:PSS, but also might promote a thinner tunneling junction overall. As a proof-of-concept, we fabricate tandem and triple-junction solar cells employing the chemically tailored EA-${\mathrm{TiO}}_{2-x}$ as n-type ICLs, and finally attain high *V*_OC_s of 1.44 V and 2.25 V, respectively.

## 2. Materials and Methods

### 2.1. Materials

For hole transport layers or ICL for holes, poly(3,4-ethylenedioxythiophene) polystyrene sulfonate (PEDOT:PSS, AI4083) was purchased from Heraeus. Chemicals for the synthesis of neat and EA-${\mathrm{TiO}}_{2-x}$ were purchased from Sigma-Aldrich: titanium tetraisopropoxide (TTIP), 2-methoxyethanol (2-ME), ethanolamine (EA), and isopropyl alcohol (IPA). Photoactive materials were either purchased from a chemical company or received from coworkers: (6,6)-phenyl C71 butyric acid methyl ester (PC_71_BM) from Nano-C, and poly[N-9′-heptadecanyl-2,7-carbazole-alt-5,5-(4′,7′-di-2-thienyl-2′,1′,3′-benzothiadiazole)] (PCDTBT) and poly[(4,4-didodecyldithieno [3,2-b:20,30-d]silole)-2,6-diyl-alt-(2,1,3-benzothiadiazole)-4,7-diyl] (SDTBT) from Bazan group and/or Heeger Center for Advanced Materials [18,19]. Other common chemicals and miscellaneous laboratory items were purchased from Sigma-Aldrich (Seoul, Republic of Korea), Alfa Aesar (Seoul, Republic of Korea), and Thermo Fisher Scientific (Seoul, Republic of Korea).

### 2.2. $TiO_{2-x}$ Synthesis and Analysis

The neat and EA-${\mathrm{TiO}}_{2-x}$ precursor solutions were synthesized with a three-neck flask equipped with a condenser, thermometer, and nitrogen (N_2_) gas purging system. For neat ${\mathrm{TiO}}_{2-x}$, 50 mL of 2-ME was injected into a N_2_-purged three-neck flask and vigorously stirred for 5 min to remove residual oxygen gas from the solvent, and 10 mL (~34 mmol) of TTIP was then added to 2-ME. After 1-min mixing of the two liquids at room temperature (RT, 25 °C), the mixture was heated up stepwise to 80 °C, maintained for 2 h, and then to 120 °C, maintained until the mixture liquid was condensed into a gel. The gel precursor was diluted with isopropyl alcohol (IPA) by 10 times and the diluted stock solution was stored in a N_2_-filled glove box. For EA-${\mathrm{TiO}}_{2-x}$ precursors, EA was added to the TTIP:2-ME mixture at room temperature. The rest of the procedures are the same as the ones for the neat ${\mathrm{TiO}}_{2-x}$. The amount of EA added to the mixture was based on mole ratios of TTIP and EA; for instance, EA-${\mathrm{TiO}}_{2-x}$ (EA:TTIP = 1:1) means that 2 mL (~34 mmol) of EA is added to the mixture of TTIP and 2-ME when 10 mL (~34 mmol) of TTIP is used. More EA is added to the mixture accordingly as the mole ratio increases.

With diluted precursor solutions (with IPA by 10 times in volume) for neat and EA-${\mathrm{TiO}}_{2-x}$, we prepared films using the drop-casting method. The prepared neat and EA-${\mathrm{TiO}}_{2-x}$ films were analyzed by XRD and FTIR. From XRD, no distinctive ${\mathrm{TiO}}_{2}$ crystal peak was found at both the neat and EA-${\mathrm{TiO}}_{2-x}$ samples, probably because the solution-processed titanium oxide films may still contain chemical substances at a relatively low processing temperature of 80 °C, resulting in an intermediate amorphous phase of titanium oxides. Upon thermal annealing at 500 °C (over 400 °C), the anatase (101) peak finally emerges at 2θ ≈ 27° for both samples (Figure S1, see in Supplementary Materials) [20]. We found that crystal peaks in the films start emerging from 400 °C, but this is not the condition in which we fabricate organic solar cells. From FTIR, we note that the films prepared at a conventional processing temperature of 80 °C show chemical residues (Figure S2, see in Supplementary Materials). For neat ${\mathrm{TiO}}_{2-x}$, symmetric and asymmetric stretching vibration peaks of the methylene groups (C–H_2_) are found at around 2850 and 2900 cm^−1^, respectively [21]. For EA-${\mathrm{TiO}}_{2-x}$, additional peaks and bands are found at around 1100, 1600, and 3100–3500 cm^−1^, respectively, accounting for C–N stretch, NH_2_ scissors band of CH_3_NH_2_, and O–H and/or N–H_2_ stretch [21,22]. Therefore, it is concluded that the neat and EA-${\mathrm{TiO}}_{2-x}$ films in this study are amorphous, and quite a lot of chemical residues still remain, particularly in the EA-${\mathrm{TiO}}_{2-x}$ film.

### 2.3. Device Fabrication

The basic structure of a single junction device architecture is as follows: glass substrate/ITO/PEDOT:PSS/photoactive layer/EA-${\mathrm{TiO}}_{2-x}$/Al (Figure S3, see in Supplementary Materials). The photoactive layer can be varied by the combination of electron donors and acceptors. For ETL, EA-${\mathrm{TiO}}_{2-x}$ (EA:TTIP = 1:1) was used. In multi-junction device architectures, ICLs are added between subcells. For instance, a tandem solar cell is composed of multiple layers on the ITO-coated glass substrate: PEDOT:PSS/photoactive layer 1/EA-${\mathrm{TiO}}_{2-x}$/PEDOT:PSS/photoactive layer 2/EA-${\mathrm{TiO}}_{2-x}$/Al. In this study, PCDTBT:PC_71_BM and SDTBT:PC_71_BM were used for photoactive layer 1 and 2, respectively. For a triple junction cell, PCDTBT:PC_71_BM was employed for photoactive layer 1 and 2 and SDTBT:PC_71_BM was used for photoactive layer 3, respectively. Detailed procedures of device fabrication can be found in our previous papers [23,24].

### 2.4. Measurements and Analysis

XRD: XRD analysis was conducted using an X-ray diffractometer (D2 PHASER, Bruker) with Cu Kα radiation (λ = 1.5405 Å) at a scan speed of 2.4° (2θ) min^−1^ over the scan range from 10° to 60° (2θ).

FTIR: Fourier transform infrared spectroscopy (FTIR) spectra were obtained using an FTIR instrument (Nicolet iS10, Thermo Fisher Scientific) coupled with a gold-coated integrating sphere (PIKE Technologies, Fitchburg) averaged over 32 scans per spectrum from 600 to 4000 wavenumbers.

J–V: *J*–*V* characteristic curves were obtained using a Keithley 2400 source measurement unit under the solar simulator equipped with an AM 1.5 filter. The light intensity was set to 100 mW cm^−2^, equivalent to 1 sun irradiance.

C–V: The capacitance–voltage (*C*–*V*) characteristics can be obtained using an Agilent 4155B semiconductor parameter analyzer. With the AC frequency set to 1 kHz with an amplitude of 70 mV peak-to-peak, a DC sweep was performed from the reverse to forward bias direction.

CPD: The contact potential difference (CPD) was obtained using a Kelvin probe (KP 6500 Digital Kelvin probe, McAllister Technical Services. Co. Ltd., Berkeley, CA, USA). To get the accurate value of work function (WF) of the stainless probe, calibration was conducted using a reference sample, highly ordered pyrolytic graphite (HOPG), the work function of which is 4.58 ± 0.03 eV. For each sample, measurements were conducted at least 100 times to get the average values of data points. The work function of a sample coated on a conducting substrate can be drawn by measuring a nulling value for a contact potential difference between a probe tip and the sample [14].

IPCE: Incident photon-to-current efficiency (IPCE) measurements were conducted by utilizing a lock-in detection system. To selectively obtain the subcell’s IPCE from a tandem solar cell, light bias was applied. To obtain the IPCE spectrum of a front cell (PCDTBT:PC_71_BM) out of a tandem solar cell, we shone a probe light modulated with an optical chopper (100 Hz) into the tandem solar cell while monochromatic lights of 532 and 700 nm were shone into the tandem solar cell to separately collect the IPCE of rear and front subcells, respectively.

## 3. Results and Discussion

**Figure 1:**
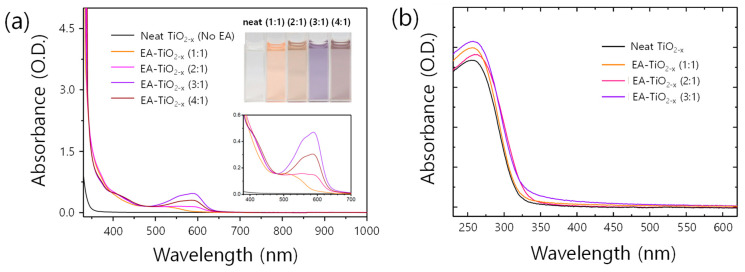
(**a**) UV-Vis-NIR absorption spectra of neat and EA-${\mathrm{TiO}}_{2-x}$ precursor solutions. The insets show the color of the solutions and a magnified shot of the spectra, where significant absorption peaks evolve. (**b**) UV-Vis absorption spectra of neat and EA-${\mathrm{TiO}}_{2-x}$ films.

Figure 1 shows different absorption spectra changed upon mole ratios of EA to TTIP. As EA increases, new peaks at around 400 nm and 550 nm occur, accounting for the color change of precursor solutions (see inset of Figure 1). There are a myriad of works on the nitrogen doping of titanium oxides in photocatalysis studies [25,26,27,28], and similar changes in spectra upon nitrogen doping are found, where new absorptions occur at around 400 and 550 nm, irrespective of doping methods with dry gases or wet chemicals [28,29]. Despite the difference in synthesis methods of nitrogen-doped titanium oxides, similar spectrum changes found in the previous works and our EA-${\mathrm{TiO}}_{2-x}$ precursor solutions may imply that the underlying physics can be commonly shared. In previous studies [25,26,30,31,32], researchers revealed that the origin of the color changes in nitrogen-doped titanium oxides are caused by the alteration of the oxidation states of titanium core ion, in turn forming new donor states in the resulting nitrogen-doped titanium oxides. Recently, Valentin and co-workers proposed theoretical models for nitrogen-doped titanium oxides by density functional theory calculations compared with experimental results [29,33,34]. For substitutional nitrogen-doped titanium oxides (i.e., O–Ti–N), N2p localized states form slightly above the valence band maximum of titanium oxides, which can be ascribed to the new absorptions in the visible range. For the interstitial nitrogen-doped titanium oxides (i.e., O–Ti–ON), two antibonding (but occupied) states lie higher than the N2p states from the substitutional nitrogen. From the theoretical models, we estimate that the new absorptions at around 400 nm and 550 nm in Figure 1 might be attributed to electronic transitions of occupied impurity states of substitutional and interstitial nitrogen atoms, respectively. While the absorption at around 400 nm newly occurs from the beginning of EA addition (EA:TTIP = 1:1) and hardly changes upon adding more EA, the absorbance at around 550 nm gradually increases upon adding more EA and reaches maximum intensity at the mole ratio of EA:TTIP = 3:1. The distinctive color features mostly disappear when the precursor solutions get a form of films (Figure 1b). In the process of spin-casting, the solutions get thinner, alcoholyzed (and/or hydrolyzed), and condensed to form gels. After gelation, the gel layer dries to form a thin solid film, losing the notable color of a precursor solution. For reference, with the precursor solution of EA:TTIP = 4:1, films were not formed on a glass substrate, so the absorption spectrum for the film sample of EA:TTIP = 4:1 is absent here.

**Figure 2:**
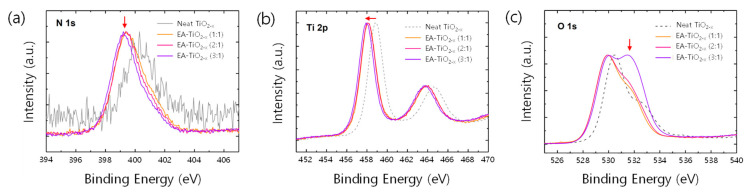
(**a**) N 1s XPS spectra, (**b**) Ti 2p XPS spectra, and (**c**) O 1s XPS spectra of neat and EA-${\mathrm{TiO}}_{2-x}$ films.

Despite the de-coloration in films, X-ray photoemission spectroscopy (XPS) spectra show distinct features, originating from nitrogen doping (Figure 2a–c). A main peak in the N 1s XPS spectrum for the neat ${\mathrm{TiO}}_{2-x}$ sample was found at around 400.5 eV (Figure 2a), which is known for chemisorbed-N_2_ molecules on ${\mathrm{TiO}}_{2}$ [30,35]. On the other hand, the N 1s XPS spectra for EA-${\mathrm{TiO}}_{2-x}$ samples can be resolved into two component peaks at around 399 eV and 400.5 eV. The peak at 399 eV is assigned to anionic N^−^ or nitrides in O–Ti–N linkages [27,34,36]. This is further supported by the noticeable binding energy (BE) shifts of Ti 2p XPS spectra. In Figure 2b, BEs of Ti 2p_3/2_ core level in EA-${\mathrm{TiO}}_{2-x}$ films are all shifted by 1 eV to the lower energy, compared to that of the neat ${\mathrm{TiO}}_{2-x}$ films. Because the electronegativity of nitrogen is lower than that of oxygen, the local electron density of Ti in the OTi–N bond can be higher than that in the Ti–O bond [26,31]. Thus, the partial electron transfer from N to Ti can cause the BE shift of the Ti2p_3/2_ core level toward lower energy in EA-${\mathrm{TiO}}_{2-x}$ samples [27,34]. Further, a noticeable change is found in O1s core level (Figure 2c) when the mole ratio of EA:TTIP increases to 3:1, where a peak centered at around 532 eV in the O1s spectrum is greatly enhanced. Since this peak (532 eV) is often observed if there is oxidized Ti–N, the EA-${\mathrm{TiO}}_{2-x}$ film prepared with the mole ratio of EA:TTIP = 3:1 contains more Ti–O–N linkages in it. Therefore, it is estimated that the EA-${\mathrm{TiO}}_{2-x}$ contains O–Ti–N and Ti–O–N (or Ti–N–O) linkages with less positive formal charges of Ti core ions in the structures.

**Figure 3:**
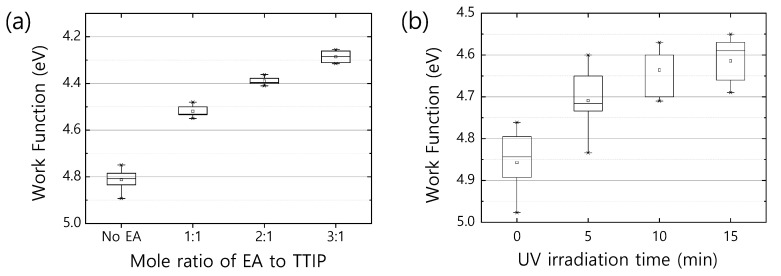
(**a**) Work function variation as mole ratio of EA to TTIP. Titanium oxide films prepared with higher mole ratio of EA to TTIP exhibit lower work function. (**b**) Work function variation of a neat ${\mathrm{TiO}}_{2-x}$ film over UV irradiation time. As UV exposure time increases, the work function of the neat ${\mathrm{TiO}}_{2-x}$ film is reduced. In the box plot, the minimum and maximum values are indicated with the asterisk (*), and small square in the box is the average value of each group.

The changes in bonding configuration surrounding the core Ti ions may make a difference in the electronic properties of EA-${\mathrm{TiO}}_{2-x}$. From a capacitance–voltage (C–V) measurement (Figure S4, see in Supplementary Materials), we noticed that the free charge carrier density increases by two orders of magnitudes as the amount of EA added to TTIP increases; the neat ${\mathrm{TiO}}_{2-x}$ exhibits $7\times 10^{16}\;{\mathrm{cm}}^{-3}$, while the EA-${\mathrm{TiO}}_{2-x}$ shows enhanced free charge carrier densities of $5\times 10^{17}\;{\mathrm{cm}}^{-3}$ for EA:TTIP = 1:1, $8\times 10^{17}\;{\mathrm{cm}}^{-3}$ for EA:TTIP = 2:1, and $5\times 10^{18}\;{\mathrm{cm}}^{-3}$ for EA:TTIP = 3:1. The enhanced free charge carrier density is evidently supported by the reduced work function of EA-${\mathrm{TiO}}_{2-x}$. Figure 3a shows the work function of neat ${\mathrm{TiO}}_{2-x}$ and EA-${\mathrm{TiO}}_{2-x}$ films, where the work function values of the samples gradually decrease from 4.8 eV to 4.3 eV as the ratio of EA:TTIP increases. Since the work function of a semiconductor can be considered equivalent to the amount of energy that an electron requires to be free with zero kinetic energy, a reduced work function implies that electrons filled up the gap states and reached higher energy levels (with respect to the vacuum level) near the conduction band edge of the metal oxide semiconductor. In most metal oxide semiconductors, gap states dominate electronic properties and are related to the imperfection of a surface or surface defects [37,38,39]. In particular, as the metal oxide semiconductors get the form of nanoparticles or an amorphous structure, surface defects predominantly affect their electronic properties. Traditionally, surface defects can be defined only at the outermost surface of a bulk crystal of a semiconductor, but semiconductors composed of nanoparticles and/or amorphous (or semicrystalline) metal oxides have much greater surface areas in the bulk solids than at their outermost surfaces. Neat ${\mathrm{TiO}}_{2-x}$ and EA-${\mathrm{TiO}}_{2-x}$ films prepared via the sol-gel process from precursor solutions take an amorphous (or semicrystalline) structure with or without nanoparticles, so the surface defects or gap states much more greatly affect their electronic properties. Typically, surface defects can trap/de-trap electrons. Vacancies and/or dangling bonds are also defects [40,41], but they can be considered electron donors. If dangling bonds or vacancies are occupied by molecules like oxygen species having high electronegativities, and/or physi-/chemi-sorbed oxygen species are attached to the surfaces, they may reduce the free charge carrier densities, capturing conducting electrons at their sites. If energy greater than their potential energy for electron capture is provided, trapped electrons can be freed out to contribute to free charge carrier densities and to result in work function reduction.

Figure 3b shows variation in the work function of neat ${\mathrm{TiO}}_{2-x}$ as a function of illumination time under UV light (365 nm). The band gap of neat ${\mathrm{TiO}}_{2-x}$ can be estimated from the UV-Vis absorption spectrum and is about 3.8 eV, so the gap states located in the band gap have potential energies lower than 3.8 eV. UV light of 365 nm is equivalent to 3.4 eV in energy, so any trapped electrons at the energy levels above 0.4 eV from the valence band edge (or oxidation potential level) of neat ${\mathrm{TiO}}_{2-x}$ can be de-trapped by the input energy of the UV light. Therefore, as the UV irradiation time increases, the work function of neat ${\mathrm{TiO}}_{2-x}$ gradually decreases. This result further implies that in EA-${\mathrm{TiO}}_{2-x}$, dangling bonds or surface defects may be preoccupied with molecules having lower electronegativity like nitrogen species such as amine groups.

Indeed, the EA-${\mathrm{TiO}}_{2-x}$ films may contain residual EA and/or amine fractions of EA. To test it, we devised a test substrate with a pH-indicator layer using the emeraldine base form of polyaniline (PANI-EB) [42]. PANI-EB is very sensitive to acids. When it contacts a trace number of protons, it immediately changes its original color (blue) to green, which is indicative of the protonation of PANI-EB. After protonation, PANI-EB is changed into the emeraldine salt form of polyaniline (PANI-ES). On the PANI-EB substrates, we deposit neat or EA-${\mathrm{TiO}}_{2-x}$. The whole structure of the test device can be found in Supplementary Materials (Figure S5a). On the test devices (Glass/PANI-EB/neat or EA-${\mathrm{TiO}}_{2-x}$), we dropped methanesulfonic acid (MSA) diluted with isopropyl alcohol (10 mg/mL) and observed how the color of the devices changed (Figure S5b, see in Supplementary Materials). It is noticeable that after MSA treatment, there is a color change in the sample with PANI-EB and neat ${\mathrm{TiO}}_{2-x}$, which means protons from MSA penetrate the titanium oxide layer and permeate the underlying PANI-EB layer, altering its original base form to salt form (PANI-ES). Interestingly, we found that the degree of color change is reduced when MSA is dropped on the samples with PANI-EB and EA-${\mathrm{TiO}}_{2-x}$. Further, the color change is almost negligible when the mole ratio of EA:TTIP is over 2:1. This implies that residual EA and/or base fraction existing in EA-${\mathrm{TiO}}_{2-x}$ films may stop the penetration of protons. If protons accumulate on the surface of EA-${\mathrm{TiO}}_{2-x}$ as a result of acid–base interaction, a very dense protonated layer may form at the interface of the EA-${\mathrm{TiO}}_{2-x}$ and MSA solution. Consequently, the positively charged surface may push back protons, blocking the further intrusion of protons into a deeper part of the layers. This basicity of EA-${\mathrm{TiO}}_{2-x}$ could help form a dipole junction when contacting acidic PEDOT:PSS at the ICJs of multi-junction solar cells, promoting a better recombination of electrons and holes at the ICJs [23,43].

**Figure 4:**
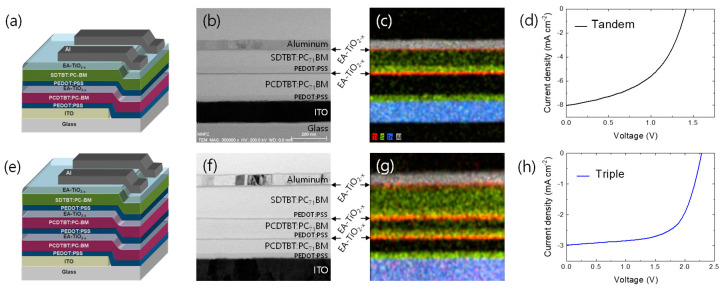
(**a**) Schematic device structure, (**b**) cross-sectional TEM image, (**c**) EDS mapping image, and (**d**) J-V characteristics of a tandem solar cell. (**e**) Schematic device structure, (**f**) cross-sectional TEM image, (**g**) EDS mapping image, and (**h**) J-V characteristics of a tiple-junction solar cell.

Using neat or EA-${\mathrm{TiO}}_{2-x}$ as an n-type ICL, we finally fabricated multi-junction solar cells. The tandem solar cell consists of Glass/ITO (80 nm)/PEDOT:PSS (25 nm)/PCDTBT:PC_71_BM (150 nm)/neat or EA-${\mathrm{TiO}}_{2-x}$ (10 nm)/PEDOT:PSS (25 nm)/SDTBT:PC_71_BM (80 nm)/EA-${\mathrm{TiO}}_{2-x}$ (10 nm)/Al (80 nm) (Figure 4a), and the triple junction solar cell is composed of Glass/ITO (80 nm)/PEDOT:PSS (25 nm)/PCDTBT:PC_71_BM (150 nm)/EA-${\mathrm{TiO}}_{2-x}$ (10 nm)/PEDOT:PSS (25 nm)/PCDTBT:PC_71_BM (150 nm)/EA-${\mathrm{TiO}}_{2-x}$ (10 nm)/PEDOT:PSS (25 nm)/SDTBT:PC_71_BM (80 nm)/EA-${\mathrm{TiO}}_{2-x}$ (10 nm)/Al (80 nm) (Figure 4e). Figure 4b,f shows cross-section images of tandem and triple junction solar cell architectures observed using transmission electron microscopy (TEM). In the TEM (Figure 4b,f) and energy dispersive X-ray (EDX) images (Figure 4c,g), each layer is clearly viewed with no intermixing. Interestingly, when we employed neat ${\mathrm{TiO}}_{2-x}$ as an n-type ICL in a tandem solar cell, the initial *V*_OC_ only shows the half-cell’s *V*_OC_ (from the rear cell), and it gradually increases upon UV irradiation and finally reaches the sum of *V*_OC_s of subcells after 30-min UV exposure (Figure S6a, see in Supplementary Materials). By contrast, as the mole ratio of EA:TTIP increases for the EA-${\mathrm{TiO}}_{2-x}$ ICL in tandem solar cells, the *V*_OC_ is gradually increased and almost reaches the full cell’s *V*_OC_ already at the mole ratio of EA:TTIP = 3:1 before UV irradiation (Figure S6b,c, see in Supplementary Materials). Using EA-${\mathrm{TiO}}_{2-x}$ ICL (EA:TTIP = 3:1) and a short UV exposure (less than 1 min), we finally achieved quite high *V*_OC_s that match the sum of *V*_OC_s of subcells (Figure 4d,h, Figures S6 and S7; and Table S1, see in Supplementary Materials), where the ohmic-like contact may be achieved via Fermi-level alignment between EA-${\mathrm{TiO}}_{2-x}$ and PEDOT:PSS ICLs of the multijunction solar cells (Figure S8, see in Supplementary Materials). In the tandem solar cell (Figure 4d), the short-circuit current density (*J*_SC_) seems to be limited by the front cell’s photocurrent at the short circuit, as shown in the incident photon-to-current efficiency (IPCE) spectrum under the light bias (Figure S7c, see in Supplementary Materials). In the triple junction solar cell, the *V*_OC_ of the multijunction solar cell shows a great match to the sum of the *V*_OC_s of subcells, while *J*_SC_ exhibits quite a low value (Figure 4h). We suspect that current mismatches between subcells may lead to such a low *J*_SC_ in the triple junction solar cell, since we used the same photoactive layer of PCDTBT:PC_71_BM for front and mid subcells. In this study, we only focused on the application of EA-${\mathrm{TiO}}_{2-x}$ to an ICL as a proof-of-concept. Further control and optimization on multijunction architectures and the combination of photoactive materials may give better performance.

## 4. Conclusions

We employed a basic amine-contained chemical (EA) as a nitrogen doping reagent to titanium oxides, and the incorporation of EA enhanced the free charge carrier density of EA-${\mathrm{TiO}}_{2-x}$ up to $5\times 10^{18}\;{\mathrm{cm}}^{-3}$ and lowered the WF to 4.3 eV. Moreover, the residual EA or amine fraction in the EA-${\mathrm{TiO}}_{2-x}$ layer may help form a dipole junction when contacting acidic PEDOT:PSS, which could promote better charge recombination at the interface of basic EA-${\mathrm{TiO}}_{2-x}$ and acidic PEDOT:PSS ICLs. Finally, using the basic EA-${\mathrm{TiO}}_{2-x}$ as an n-type ICL paired with the acidic PEDOT:PSS ICL, we fabricated tandem and triple junction solar cells with high *V*_OC_s of 1.44 V and 2.25 V, respectively.

## Data Availability

The data presented in this study are available on request from the corresponding authors.

## Supplementary Materials

The following supporting information can be downloaded at: https://www.mdpi.com/article/10.3390/polym16050595/s1, Figure S1: XRD spectra of (a) neat TiO_2−x_ coated glass substrate, and (b) EA-TiO_2−x_ coated glass substrate treated with different temperature (80 °C, and 500 °C). XRD spectrum of bare glass substrate is provided as a baseline; Figure S2: FTIR spectra of bare glass substrate, neat TiO_2−x_ coated glass substrate, and EA-TiO_2−x_ coated glass substrate; Figure S3: Device structures of (a, b) single-junction solar cells with the different photoactive layer of SDTBT:PC71BM and PCDTBT:PC71BM, respectively; Figure S4: Mott–Schottky plots of neat-TiO_2_-x and EA-TiO_2−x_ films. From the slopes of the curves, free charge carrier densities were estimated; Figure S5: (a) A device indicating pH. Upon acid penetration, the device changes its original color (blue) to green as the underneath PANI-EB layer changes its base form into the salt form, PANI-ES. (b) UV-Vis spectra of the device upon exposure of diluted MSA indicate color changes as the absorption band in the range of 600 - 800 nm evolves in the neat and EA-TiO_2−x_ (1:1) samples. In contrast, very little changes in color and spectrum are witnessed in the EA-TiO_2−x_ samples with higher mole ratios of EA:TTIP = 2:1; Figure S6: (a) J-V characteristics of a tandem solar cell with neat TiO2-x, (b) J-V characteristics of a tandem solar cell with EA-TiO_2−x_ (EA:TTIP = 3:1), (c) Semi-log plots of J-V characteristic curves of tandem solar cells with neat TiO_2−x_ and EA-TiO_2−x_ with different mole ratio of EA and TTIP indicate that VOC is gradually enhanced as the mole ratio of EA and TTIP increases; Figure S7: (a) J-V characteristics of front and rear cells composed of PCDTBT:PC71BM and SDTBT:PC71BM, respectively. (b) UV-Vis absorption spectra of PCDTBT and SDTBT. (C) IPCE spectra for front and rear cells of a tandem solar cell. The selected wavelengths (700 nm for the front cell’s IPCE and 532 nm for the rear cell’s IPCE) of light were shone onto the tandem solar cell to separately obtain each subcell’s IPCE; Figure S8: (a) Energy band diagram of a tandem solar cell. (b) Forming Ohmic-like junction in between subcells via Fermi-level alignment between n-type EA-TiO_2−x_ and p-type PEDOT:PSS ICLs of multijunction solar cells; Table S1: Solar cell parameters for front, rear and multijunction solar cells are summarized.
