# Supplementary material for: Chemically Engineered Titanium Oxide Interconnecting Layer for Multijunction Polymer Solar Cells
Source: Polymers (Basel). 2024 Feb 21;16(5):595. doi: 10.3390/polym16050595 (PMC10935189; doi:10.3390/polym16050595)
Supplement: Supplementary file 1 [file polymers-16-00595-s001.zip › polymers-2860317-supplementary.pdf]

Supplementary Materials: Chemically Engineered Titanium Oxide Interconnecting Layer for Multijunction Polymer Solar Cells

Geunjin Kim, Hyungcheol Back, Jaemin Kong , Laiba Naseer, Jiwon Jeong, Jaehyoung Son, Jongjin Lee, Sung-Oong Kang and Kwanghee Lee

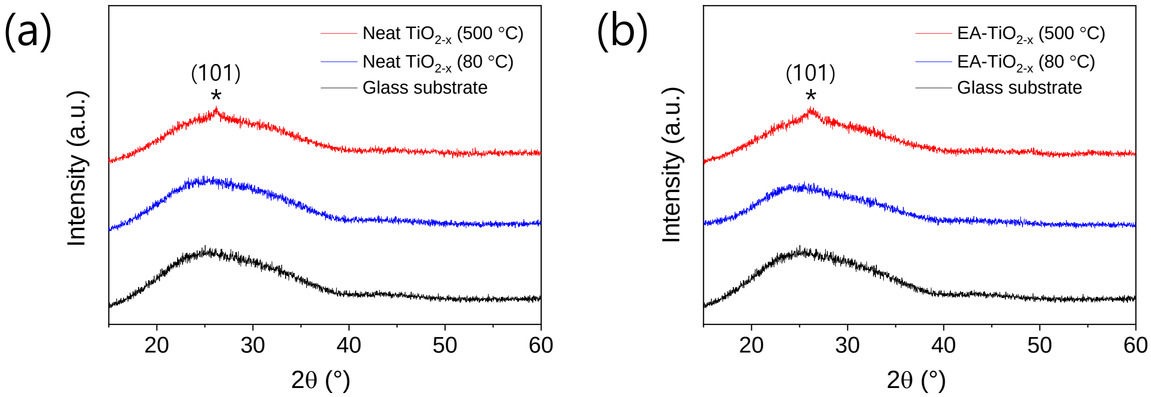

Figure S1. XRD spectra of (a) neat TiO_{2-x} coated glass substrate, and (b) EA-TiO_{2-x} coated glass substrate treated with different temperature (80 °C, and 500 °C). XRD spectrum of bare glass substrate is provided as a baseline.

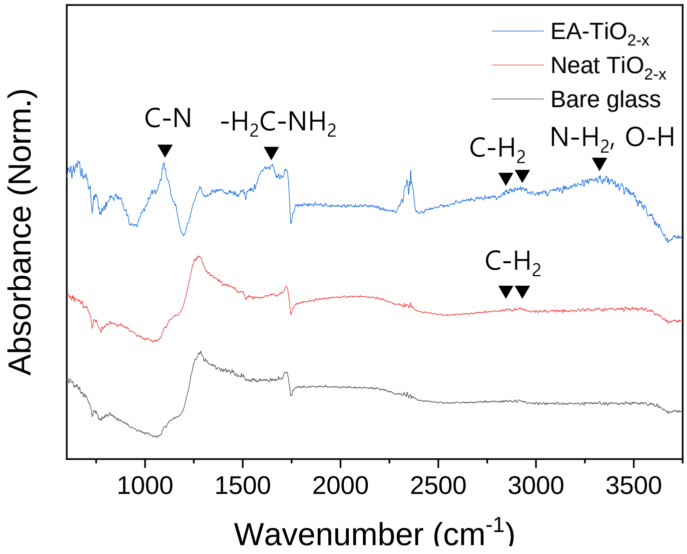

Figure S2. FTIR spectra of bare glass substrate, neat TiO_{2-x} coated glass substrate, and EA-TiO_{2-x} coated glass substrate.

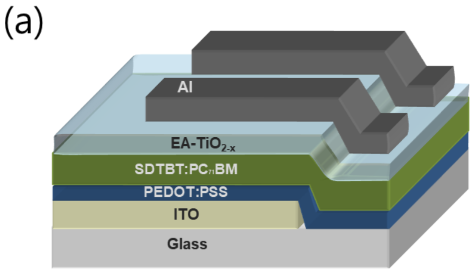

Figure S3. Device structures of (a, b) single-junction solar cells with the different photoactive layer of SDTBT:$PC_{71}BM$ and PCDTBT:$PC_{71}BM$, respectively.

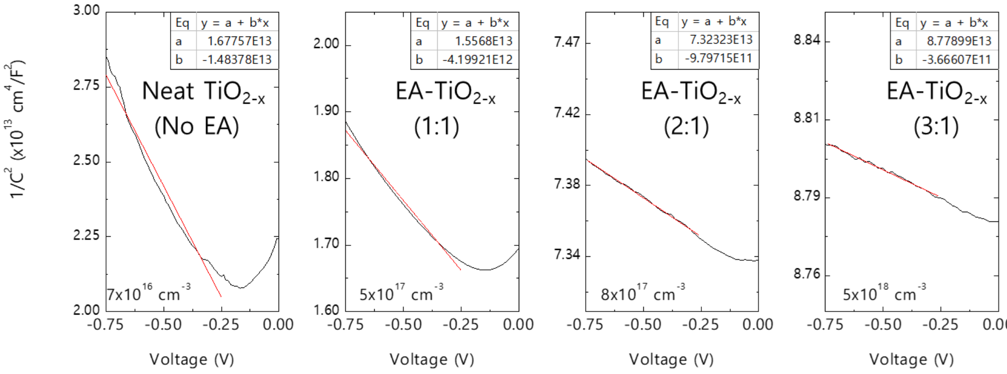

Figure S4. Mott–Schottky plots of neat-TiO_{2-x} and EA-TiO_{2-x} films. From the slopes of the curves, free charge carrier densities were estimated.

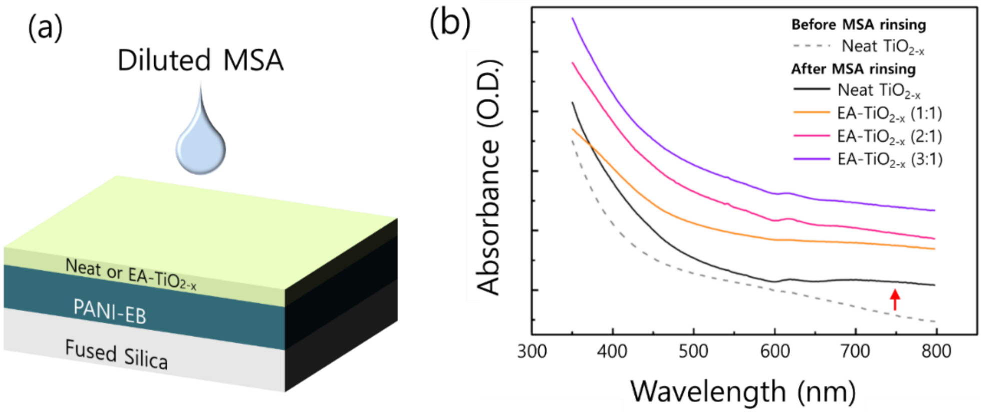

Figure S5. (a) A device indicating pH. Upon acid penetration, the device changes its original color (blue) to green as the underneath PANI-EB layer changes its base form into the salt form, PANI-ES. (b) UV-Vis spectra of the device upon exposure of diluted MSA indicate color changes as the absorption band in the range of 600 - 800 nm evolves in the neat and EA-TiO_{2-x} (1:1) samples. In contrast, very little changes in color and spectrum are witnessed in the EA-TiO_{2-x} samples with higher mole ratios of EA:TTIP = 2:1.

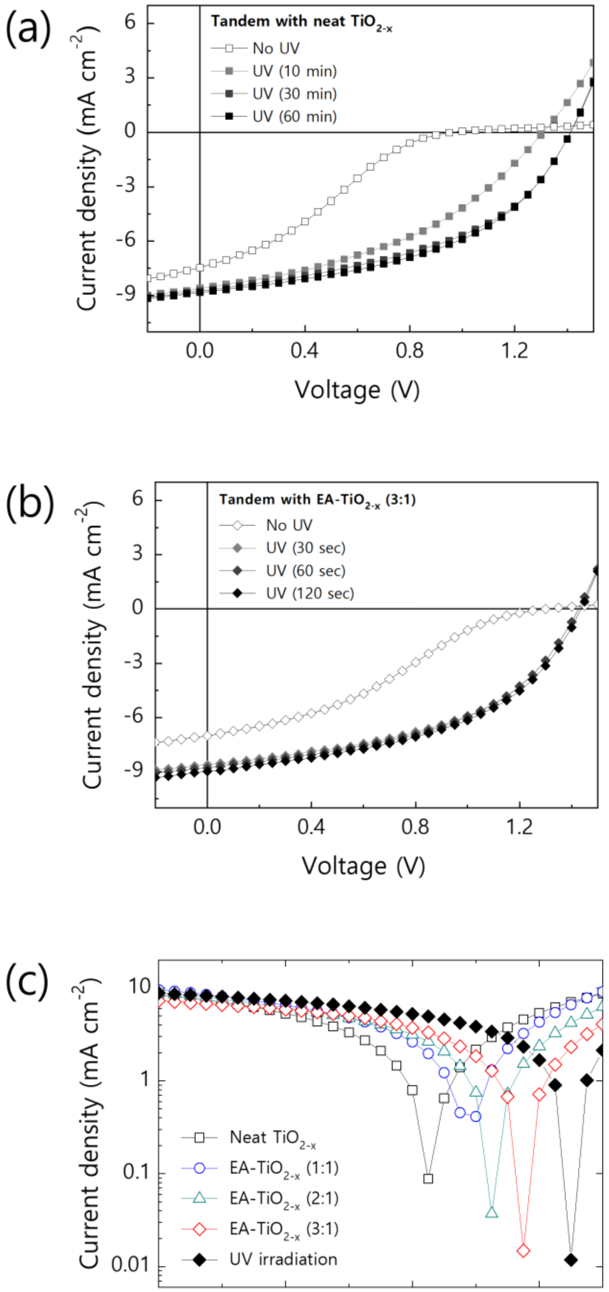

Figure S6. (a) J-V characteristics of a tandem solar cell with neat TiO_{2-x}, (b) *J-V* characteristics of a tandem solar cell with EA-TiO_{2-x} (EA:TTIP = 3:1), (c) Semi-log plots of *J-V* characteristic curves of tandem solar cells with neat TiO_{2-x} and EA-TiO_{2-x} with different mole ratio of EA and TTIP indicate that V_{OC} is gradually enhanced as the mole ratio of EA and TTIP increases.

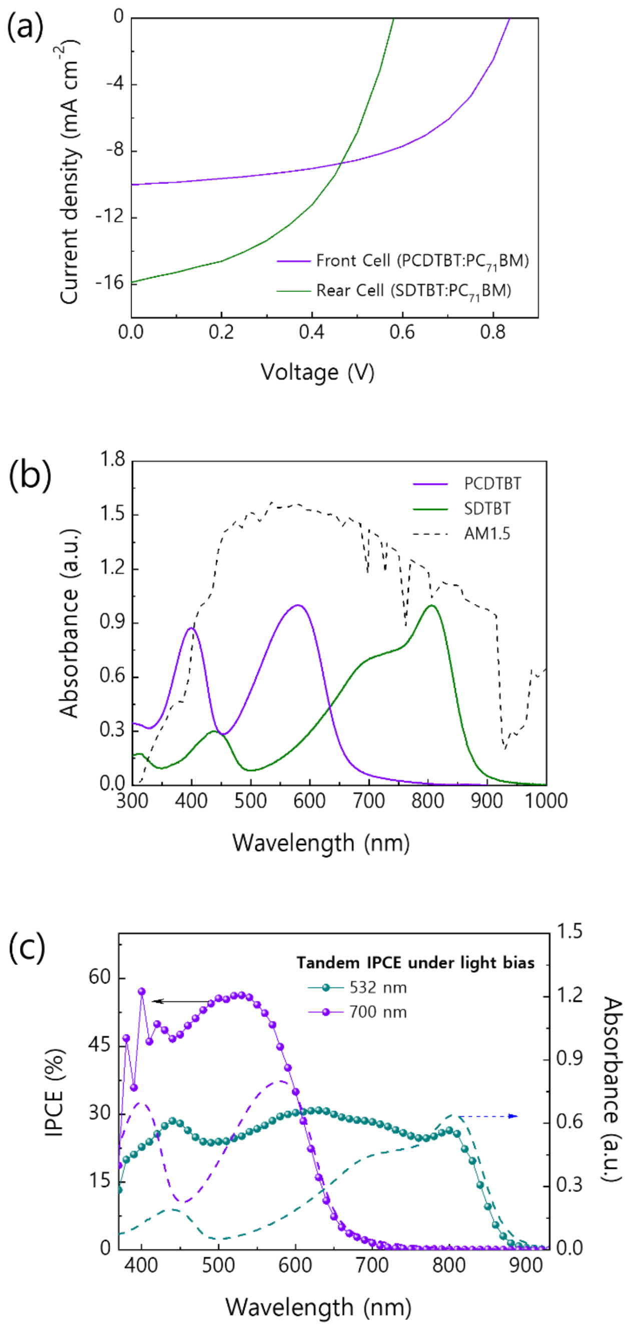

Figure S7. (a) J-V characteristics of front and rear cells composed of PCDTBT:$PC_{71}BM$ and SDTBT:PC71BM, respectively. (b) UV-Vis absorption spectra of PCDTBT and SDTBT. (C) IPCE spectra for front and rear cells of a tandem solar cell. The selected wavelengths (700 nm for the front cell's IPCE and 532 nm for the rear cell's IPCE) of light were shone onto the tandem solar cell to separately obtain each subcell's IPCE.

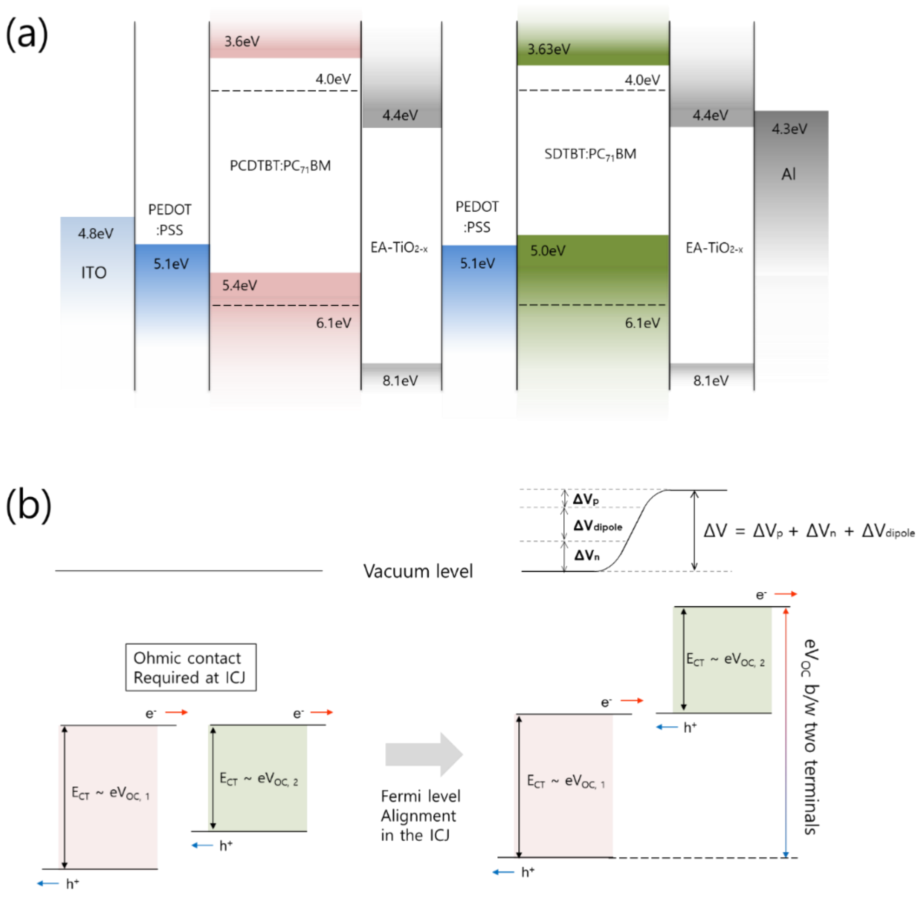

Figure S8. (a) Energy band diagram of a tandem solar cell. (b) Forming Ohmic-like junction in between subcells via Fermi-level alignment between n-type EA-TiO_{2-x} and p-type PEDOT:PSS ICLs of multijunction solar cells.

Table S1. Solar cell parameters for front, rear and multijunction solar cells are summarized.

	Front (PCDTBT:PC_{71}BM)	Rear (SDTBT:PC_{71}BM)	Tandem	Triple
V_{OC} (V)	0.85	0.58	1.44	2.25
J_{SC} (mA cm^{-2})	10.0	15.9	8.9	2.96
FF	0.52	0.49	0.48	0.66
PCE (%)	4.42	4.50	6.15	4.40
